# Effects of stress hormones on the brain and cognition: Evidence from normal to pathological aging

**DOI:** 10.1590/S1980-57642011DN05010003

**Published:** 2011

**Authors:** Juliana Nery de Souza-Talarico, Marie-France Marin, Shireen Sindi, Sonia J. Lupien

**Affiliations:** 1 PhD, Department of Medical-Surgical Nursing, School of Nursing, University of São Paulo, São Paulo SP, Brazil and Behavioral and Cognitive Neurology Unit, Department of Neurology, Faculty of Medicine, University of São Paulo, São Paulo SP, Brazil.; 2 MSc, Center for Studies on Human Stress, Mental Health Research Center Fernand-Seguin, Louis-H. Lafontaine Hospital, Université de Montreal, Canada.; 3 MSc, Department of Neurology and Neurosurgery, McGill University, Montreal, Canada.; 4 PhD, Center for Studies on Human Stress, Mental Health Research Center Fernand-Seguin, Louis-H. Lafontaine Hospital, Université de Montreal, Canada.

**Keywords:** glucocorticoids, memory, aging, Alzheimer’s disease

## Abstract

Several studies have demonstrated a wide cognitive variability among aged individuals. One factor thought to be associated with this heterogeneity is exposure to chronic stress throughout life. Animal and human evidence demonstrates that glucocorticoids (GCs), the main class of stress hormones, are strongly linked to memory performance whereby elevated GC levels are associated with memory performance decline in both normal and pathological cognitive aging. Accordingly, it is believed that GCs may increase the brain’s vulnerability to the effects of internal and external insults, and thus may play a role in the development of age-related cognitive disorders such as Alzheimer’s disease (AD). The aim of this review article was to investigate the effects of GCs on normal and pathological cognitive aging by showing how these hormones interact with different brain structures involved in cognitive abilities, subsequently worsen memory performance, and increase the risk for developing dementia.

## Abstract (pt)

Vários estudos têm demonstrado uma ampla variabilidade cognitiva entre indivíduos idosos. Um dos fatores que tem sido associado com esta heterogeneidade é a exposição crônica ao estresse ao longo da vida. Evidências em humanos em animais têm mostrado que os glicocorticóides (GCs), principal classe de hormônios do estresse, estão fortemente associados com o desempenho da memória, sendo que concentrações elevadas de GCs está correlacionada com declínio da memória no envelhecimento cognitivo normal e patológico. Consequentemente, alguns autores têm proposto que os GCs podem aumentar a vulnerabilidade do cérebro aos efeitos de insultos internos e externos desempenhando, portanto, papel importante no desenvolvimento de transtornos cognitivos associados à idade como a doença de Alzheimer (AD). Este artigo de revisão discute os efeitos dos GCs no envelhecimento cognitivo normal e patológico demonstrando como estes hormônios interagem com diferentes estruturas cerebrais envolvidas em habilidades cognitivas e subsequentemente pioram o desempenho da memória e aumentam o risco para o desenvolvimento de demência.

It is well-documented in the scientific literature that older adults show significant variability in terms of cognitive performance. Consequently, some researchers became particularly interested in understanding the various factors that could contribute to this differential aging phenomenon. Differing patterns of glucocorticoids (GCs) secretion have also been reported in older adults, with some individuals attaining very high levels of GCs, while others maintain moderate levels. Hence, it has been proposed that the cognitive variability observed in older adults could be explained by exposure to elevated levels of GCs throughout the life span.

Glucocorticoids are a major class of stress hormones released by activation of the hypothalamic-pituitary-adrenal (HPA) axis. When an organism is exposed to a stressful situation, the HPA axis is activated. This cascade is first initiated by the release of corticotropin releasing factor (CRF) from the paraventricular nucleus of the hypothalamus. This leads to the secretion of adrenocorticotropin hormone (ACTH) from the pituitary and the release of GCs (mainly corticosterone in animals and cortisol in humans) from the adrenal glands then ensues. It is important to note that basal GC secretion follows a circadian rhythm characterized by a peak reached approximately 30 to 60 minutes after awakening followed by a progressive decline throughout the day.

Following an increase in GCs, the organism needs to return to a homeostatic basal state. In order to do so, GCs cross the blood-brain-barrier exploiting their liposoluble properties, and bind to the pituitary and the hypothalamic regions to exert negative feedback. Importantly, other brain structures are rich in GC receptors. There are two types of GC receptor: mineralocorticoid receptors (MR or Type I) and glucocorticoid receptors (GR or Type II). They differ from each other with respect to their affinity and their distribution throughout the various brain structures. GCs bind with a much higher affinity to MRs than GRs.^1^ This means that in the morning period, GCs occupy more than 90% of MRs, but only 10% of GRs. However, when facing a stressor and/or during the circadian peak of GC secretion, MRs are saturated and approximately 70% of the GRs are occupied.^2^ Moreover, the two categories of GC receptors differ with regards to their distribution in the brain. In fact, the MRs are exclusively present in the limbic system whereas GRs are present in both subcortical and cortical structures, with a preferential distribution in the prefrontal cortex.^3^

Briefly, GC receptors are mainly found in the following regions: the amygdala, prefrontal cortex and hippocampus. Given that the amygdala is very important for processing emotional information, the prefrontal cortex is involved in executive functions, and that the hippocampus is well-known for its role in learning and memory, this represented a good rationale for scientists to further investigate the role of stress and stress hormones on the different cognitive functions subserved by these brain regions. Interestingly, the effects of GCs on cognitive performance are quite variable and depend on many factors, one of them being the duration of exposure to high levels of GCs. Thus, acute effects of GCs on memory are quite distinct from chronic effects.

## GCs and memory: acute effects

Acute variations in GC levels, induced either endogenously in response to a stressor or exogenously with pharmacological protocols, profoundly impact memory performance. These effects are differential and depend on multiple factors, such as time of day and the population being studied. Notwithstanding these two modulators, one of the most important factors that needs to be taken into account is the memory process (e.g. consolidation vs. retrieval) being studied.

**GCs and memory consolidation** – Memory consolidation refers to the process whereby newly acquired information is transferred from an initially unstable state in the short-term memory system into a stable state in the long-term memory system.^4^ This process of memory consolidation can be modulated (enhanced or impaired) by different manipulations administered proximally to the time of encoding, which demonstrates the unstable nature of the memory trace early in the consolidation process. Additionally, GCs have the capacity to modulate this consolidation process. In general, an elevation in GC levels enhances memory consolidation.^5,6^ Moreover, it is important to note that very low levels of GCs (induced pharmacologically by the administration of metyrapone which blocks the synthesis of GCs) can impair the consolidation of both neutral and emotional information.^7^

**GCs and memory retrieval** – Memory retrieval refers to the notion of remembering memory traces that are already consolidated. Interestingly, the impact of GCs on memory retrieval is the opposite from that observed on the consolidation process, whereby an elevation in GC levels can impair the process of memory retrieval.^8-10^ In other words, when GC levels are elevated, the capacity to retrieve a previously consolidated memory trace is reduced.^10^

However, it would be wrong to assume that the relationship between GCs and memory retrieval is linear. Studies have demonstrated that low GC levels, induced pharmacologically, are also harmful for delayed memory recall.^11^ These findings are in line with the proposed inverted-U shape relationship between circulating levels of GCs and memory performance.^12^ In this formulation, both very low and very high levels of GCs are detrimental for memory performance, whereas moderate levels result in optimal performance. This can be explained by the occupancy of GC receptors and has been termed the GC Receptor Balance Hypothesis. Specifically, it proposes that when the MR/GR ratio is high (low or moderate levels of GCs, thus high occupation of MRs but low occupation of GRs), memory performance is enhanced. However, when the MR/GR ratio is low (high levels of GCs, thus high occupation of MRs and GRs), memory performance is impaired. Supporting this hypothesis, a recent finding demonstrated an association between impaired free recall and low glucocorticoids levels induced by administration of cortisol synthesis inhibitor. However, recognition remained unaffected.^104^

**GCs and working memory** – For many years, the effects of GCs on memory were thought to be restricted to declarative memory, which is subserved by the hippocampus. In 2000, a study on primates demonstrated the presence of GC receptors in the prefrontal cortex (mainly Type II receptors), which led scientists to hypothesize that an increase in GC levels could have an impact on cognitive functions which are dependent on the frontal lobes. Various neuropsychological studies have demonstrated the role of the prefrontal cortex in working memory, the process allowing an individual to maintain a limited amount of information online for a short amount of time.^13^

With this in mind, the effects of GCs on working memory have been investigated in different studies. For example, it has been shown that hydrocortisone administration had detrimental effects on working memory in young healthy adults.^14^ Interestingly enough, declarative memory was also measured in the same study and was unaffected by the hydrocortisone administration. This demonstrates not only that working memory is impaired by an elevation in GC levels but also that this cognitive function is more sensitive to GC variations compared to declarative memory. Other studies using psychosocial stressor instead of hydrocortisone administration have also reported impairments in working memory functions.^15^

Clearly, acute modulation of GC levels could have significant effects on different memory processes. Moreover, as mentioned previously, older adults as a population demonstrate broad variability in terms of cognitive performance.^101,102^ Based on animal studies supporting the relationship between elevated levels of GCs and memory performance in aged rodents, some researchers have started to question whether the cognitive impairments observed in a certain portion of older adults might be explained by their long-term exposition to elevated levels of GCs.

## Aging and GCs

Older adults demonstrate great variability in HPA axis activity and in its impact on cognitive performance. Research findings on stress and aging are inconclusive as to whether basal GC levels increase during the aging process. Whereas some studies on older adults yield evidence for elevated basal levels of cortisol,^16,17^ others have shown lower basal levels,^18,19^ and some evidence suggests that cortisol levels remain stable in healthy older subjects.^20,21^ Additionally, circadian rhythm among older adults also tends be characterized by an advanced phase.^16^ This finding revealed that the diurnal rhythmicity of cortisol secretion is preserved in old age, but the relative amplitude was dampened, and the timing of the circadian elevation was advanced.^16^ Further, a study on the circadian rhythm cortisol profile in a large community dwelling population showed that those with a raised cortisol profile tended to be older than those with normative curves.^103^ Different sampling methods as well as times at which samples were collected may partly explain the diverse results obtained thus far. Moreover, inter-individual differences play an important role in determining cortisol levels. Lupien et al. (1996) conducted a longitudinal study where a sample of healthy older adults had cortisol levels measured on an annual basis. Their findings demonstrated that basal cortisol levels could be categorized into three sub-groups.^22^ One group showed an annual increase and had high current levels (Increasing / High group), another had an annual increase with moderate current levels (Increasing / Moderate group), while the final group presented with an annual decrease and moderate current cortisol levels (Decreasing / Moderate group). This evidence suggests that HPA-axis functioning displays vast inter-individual variation among older adults. Interestingly, the group with high current levels and an annual increase showed impaired cognitive performance compared to the other groups. Higher levels of basal GCs may not be a feature of normal aging but instead serve as a marker for cognitive impairment and pathological aging.^23^

Perhaps aging does not have an impact on the regulation of basal HPA functioning, but instead may have an impact on HPA reactivity. Older adults show an altered HPA recovery response to both psychological and pharmacological challenges.^24,25^ Evidence suggests that older individuals tend to show decreased responsiveness to HPA negative feedback inhibition, as demonstrated by a blunted suppression of plasma ACTH in response to ACTH challenge.^24,26^ Insufficient sensitivity to cortisol feedback inhibition is associated with memory impairments.^27^ Moreover, prolonged increases in levels of cortisol reactivity were observed in response to a driving simulation test.^28^ Consistently, some studies have indicated that older adults show large cortisol reactivity in response to a psychosocial stressor (which involved a public speaking task in a laboratory setting),^29^ yet conversely other studies have found a decreased response.^30^ More recent findings failed to detect a correlation between age and cortisol reactivity in response to a psychosocial stress task.^31^ Such results may provide further evidence for the inter-individual variation among older populations. Notably, socio-economic characteristics such as education level and social economic status may also contribute to contradictory findings. Recently, educational level has been reported as a factor that may modulate cortisol reactivity to psychosocial stress in aging.^100^ The cited study demonstrated that elderly participants with a low educational level showed greater specific stress response to the Trier Social Stress Test.^100^

**Aging, GCs, memory and the hippocampus** – In aged rodents, chronic stress and high levels of basal GC were associated with impaired cognitive performance on hippocampal-dependent tasks, as well as decreased hippocampal volume, hippocampal neuronal loss and dendritic atrophy.^32-39^ Interestingly, prospective reports of high levels of chronic stress over a period of 20-years were associated with hippocampal atrophy and reduced orbitofrontal cortex grey matter.^40^ Impairments have been observed in hippocampal dependent tasks including spatial memory.^33,35^ Chronically high levels of GC attenuate neurogenesis in the dentate gyrus region of the hippocampus, a brain region that continues to generate neurons throughout the adult lifespan.^41^ Intriguingly, if GC secretion is decreased from midlife onwards, increased neurogenesis and preserved spatial memory functioning is subsequently observed in aging.^42^ This evidence suggests that neuronal plasticity may play an important role in maintaining cognitive functioning during aging.

When middle-aged rats are administered high levels of GC for extended periods of time, the resulting deficits in memory performance are similar to those found in aged rats with high basal GC levels.^43^ Conversely, memory impairments and hippocampal atrophy are not present when cortisol levels are maintained at low levels (either through surgical andrenalectomy or pharmacologic methods).^43^ Consistent discoveries have formed the foundations of the ‘glucocorticoid cascade hypothesis',^44^ which postulates that exposure to elevated levels of GC for extended periods of time can have a cumulative impact, which in turn increases the risk for memory impairments and hippocampal atrophy. According to this hypothesis, hippocampus dysfunction disrupts the normal negative feedback to the HPA axis, which may result in hypercortisolemia. Prolonged hypercortisolemia may also lead to hippocampal dysfunction, potentially creating a vicious circle.^44^ Based on more recent evidence regarding potential underlying mechanisms of this relationship, this hypothesis is currently referred to as the ‘neurotoxicity hypothesis’.

As previously stated, cognitive performance and hippocampal functioning both show significant inter-individual variation among older adults.^45,46^ Findings from a recent study demonstrated that hippocampal volume also shows a considerable degree of variability among older adults.^47^ Reduced hippocampal volume may be a marker of cognitive decline as opposed to a natural consequence of healthy aging.^48^

Consistently, prospective studies have demonstrated that older adults with increased levels of cortisol presented deficits in memory performance compared to groups with stable cortisol levels.^49,50^ More significant increases in cortisol reactivity to a psychosocial stressor were associated with deficits in declarative memory.^51^ Similarly, longitudinal data has shown elevated levels of cortisol to be correlated with impaired memory performance and reduced hippocampal volume in a sample of healthy older adults.^52^ These findings are consistent with the neurotoxicity framework previously described.

One factor that may partially contribute to the high variability in cognitive performance among elderly subjects is the testing environment that may itself be inherently stressful. When measuring cognitive performance among older individuals, the testing environment is a critical factor to take into account. It has been demonstrated that older adults are more reactive to the environment in which they are tested compared to young adults.^53,54^ Older adults show higher levels of cortisol at the time of their arrival at the laboratory to perform tests of cognitive functioning.^53^ However, after memory testing and performance of a psychosocial stress task, older adults’ cortisol levels no longer differ from young adults, elucidating their heightened sensitivity to the testing environment. Moreover, when older adults perform cognitive tasks that emphasize the memory component, they show impaired memory performance compared to young adults. Yet when the task instructions (for the same tasks) are altered to decrease the emphasis on the memory, performance between older and young adults becomes quite similar.^55,56^ It is possible that having cognitive capacities tested is a stressful task for older adults due to their concerns about symptoms of dementia. Clearly, a stressful testing environment for cognitive testing may play an important role in predicting stress-induced memory impairments observed among older adults. Stereotypes in aging and memory loss may also have an important role to play in influencing older adults’ beliefs regarding their cognitive capacities.^57^ Evidently, the testing environment consists of a variety of components that need to be adjusted for older adults in order to prevent stress-induced deficits in memory performance.

The evidence on the relationship between glucocorticoids and memory decline allied to hippocampal atrophy has aroused the interest of different research groups in investigating the involvement of stress hormones in pathological cognitive impairment among the elderly.

## Acute and chronic stress in Alzheimer dementia-type

The neurotoxicity hypothesis previously described has given rise to the investigation of the relationship between stress hormones and Alzheimer’s disease (AD), a neurodegenerative disorder clinically characterized by progressive cognitive and functional impairments. In line with this hypothesis, it has been proposed that the hippocampal atrophy reported in AD subjects may lead to negative feedback caused by HPA axis dysfunction that produces high levels of cortisol. Such dysregulation may be detrimental to hippocampal neurons and thereby compromise memory performance.^2,44^ Accordingly, transgenic rodent models of AD have demonstrated increased levels of corticosterone under acute and chronic stress situations.^58-60^ In humans, recent findings have demonstrated a significant association between decreased visuospatial memory, decreased hippocampal CA1 volume (hippocampal region which expresses high levels of GR receptors) and abnormal negative feedback in the HPA axis among patients with mild to moderate AD.^61^ Further, several studies have shown that subjects with AD^61-70^ and Mild Cognitive Impairment (MCI), a state between normal aging and dementia,^71^ have higher basal cortisol levels than healthy elderly individuals.^70,72,73^ However, longitudinal findings failed to demonstrate significant differences in cortisol levels between AD and healthy controls.^66^ Notably, biological specimens as well as time and methods of sampling may contribute to contradictory findings. Recently, seasonal variations in cortisol levels have been reported as a factor influencing GC concentrations in MCI and AD subjects.^70^ The study reveals that controlling for seasonal effects on basal salivary cortisol levels, participants with MCI and AD secreted higher cortisol concentrations compared to healthy elderly controls.^70^ In addition to the neurotoxicity hypothesis as the central mechanism that induces GCs elevation, exposure to stressful events has also been associated with increased AD development. Epidemiological evidence has shown that older adults who are more prone to distress are 2.7 times more likely to develop AD compared to those who are not prone to distress.^74^ Moreover, high levels of perceived stress have been associated with elevated cortisol levels in MCI subjects.^75^

As shown in normal cognitive aging, recent animal and human evidence has demonstrated a relationship between GCs and memory performance in pathological cognitive conditions. In experimental models of AD, both acute and chronic exposure to stress was followed by further declines in cognitive function.^76,77^ Similarly, increased levels of basal plasma cortisol were inversely related to cognitive performance in both MCI^68,78^ and AD patients.^86^ Moreover, a positive association between high cortisol levels and disease progression was reported in AD subjects.^69^

Despite the evidence of a relationship between cortisol levels and cognitive impairment, the role of GCs in the neuropathology of AD remains elusive. In fact, beyond the association between elevated cortisol levels and AD, recent studies have shown that GCs exacerbate AD pathogenesis and may worsen cognitive deficits.^79^

The neuropathology of AD is characterized by extracellular plaque formation by β-amyloid (A*β*) deposition and intracellular neurofibrillary tangles by aggregates of protein tau hyperphosphorylation in the cortex and hippocampus.^80^ Alterations in hippocampus plasticity and neurogenesis have also been reported in AD subjects.^81,82^

Besides previous animal studies showing that accumulations of A*β* in the hippocampus precede increases in corticosterone,^58,60,83^ elevations in GCs have also been associated with both accelerated A*β* production and decreased A*β* degradation in AD.^84^ Taken together, these findings support the hypothesis that GCs may play a role in AD neuropathology. Accordingly, increase in A*β* production can be pharmacologically reversed after administration of metyrapone in animals submitted to chronic restraint stress leading to a decrease in A*β* deposition.^84^ Interestingly, better spatial working memory was also observed after metyrapone administration in unstressed rodent models of AD,^83^ implying that GCs may also contribute to the cognitive impairments exhibited by individuals with dementia.

Increased corticosterone levels induced by chronic immobilization stress or by acute administration of dexamethasone are associated with both accelerated A*β* plaque formation and tau protein phosphorylation, once again showing a relationship between AD neuropathology biomarkers and stress hormones.^59,84^ Additionally, recent evidence links GCs, A*β* formation and neuronal apoptosis after chronic administration of high doses of dexamethasone in mice models of AD.^85^

Although evidence suggests a relationship between GCs and AD neuropathology biomarkers, the exact mechanisms involved in this association are unclear. It is known that oxidative stress (imbalance between reactive oxygen species production and antioxidant defenses) and hippocampal dysfunction can be observed in both stressed and AD participants. This could represent a potential pathway in which stress hormones play a role in AD pathogenesis.

In agreement with this, chronic restraint stress, sleep deprivation and social isolation are associated with markers of oxidative stress in the brain.^86-88^ In fact, GCs have been proposed to be a marker of susceptibility to oxidative brain damage since rodents exposed to acute stress have shown accumulation of oxidative/nitrosative and pro-inflammatory mediators in the brain while showing less anti-inflammatory protection.^89^ Some studies have shown that A*β* deposition induces formation of reactive oxygen species leading to lipid peroxidation and protein oxidation^90,91^ in MCI and AD subjects. Oxidative stress markers have been shown to precede increases in AD neuropathological hallmarks,^92^ suggesting that oxidative stress may contribute to AD development. Concordantly, previous associations between AD pathology and common oxidized brain proteins in MCI subjects, early AD (EAD) and late-stage AD^91,93^ have been substantiated. The presence of the epsilon^4^ allele of apolipoprotein E (APOE), a risk factor for sporadic AD, has also been associated with nitrite oxidative stress markers.^94^ Interestingly, recent findings have shown that increases in lipid peroxidation were three-fold greater in stressed AD mice compared to unstressed controls, and these elevations in oxidative stress markers decrease after pharmacological blocking of corticosterone.^84^

Regarding hippocampal plasticity, chronic adverse stress may lead to increased apoptosis of newly generated neurons in the hippocampus, a group of cells believed to be associated with hippocampus plasticity and neurogenesis.^95^ Further, elevated GC levels were found to lead to atrophy of the CA^3^ apical dendrites region of the hippocampus in rodents.^96^ Intriguingly, a similar reduction in dendritic spine density is observed in AD,^97^ and previous studies have shown a relationship between hippocampal atrophy and elevated GC levels in animals and humans.^98,99^ This association among stress hormones, hippocampal cell loss and plasticity reveal another common finding observed in chronic stress and AD pathology. In line with these findings, it has been proposed that the GCs released in response to a psychological stressor trigger oxidative stress markers, which increase the susceptibility of the brain to the damaging effects of pathological aging.

## Conclusion

The literature has extensively shown that elderly subjects are widely exposed to adverse and challenging situations in their daily routine and are consequently also exposed to stress hormones and their effects on cognition. In addition to age-induced brain modifications, these individuals carry the effects of stress hormones cumulated throughout life, which may increase their susceptibility to pathological cognitive impairment. Although the exact mechanisms by which stress hormones may contribute to dementia development are not fully understood, the literature has consistently shown a strong association between GCs and biomarkers of AD pathogenesis during aging. Hence, psychoneuroendocrine evaluation with appropriate methods allied to neuropsychological assessment may represent an additional factor to be taken into account in the AD screening. In addition, given that psychological stress can be managed to a certain extent, learning how to decrease stress levels using evidence-based stress management strategies may be beneficial for older adults. Findings in elderly individuals may set the stage for more complex models that can help prevent the development of pathological cognitive impairment.
